# Incorporation of β-actin loading control into zymography

**DOI:** 10.14440/jbm.2016.157

**Published:** 2016-12-06

**Authors:** Natasha Govindasamy, MengJie Yan, Paul Jurasz

**Affiliations:** ^1^Department of Pharmacology, Faculty of Medicine and Dentistry;; ^2^Cardiovascular Research Centre;; ^3^Faculty of Pharmacy and Pharmaceutical Sciences; and; ^4^Mazankowski Alberta Heart Institute, University of Alberta, 3-142 E Katz Group-Rexall Centre for Pharmacy & Health Research, 11361-87 Avenue, Edmonton, AB, Canada, T6G 2 E1

**Keywords:** matrix metalloproteinase, zymography, immunoblot, β-actin, loading control

## Abstract

Gelatin zymography and immunoblot are widely used gel electrophoresis techniques to study matrix metalloproteinases-2 and -9. Each method has its advantages and disadvantages. Zymography is exquisitely sensitive but offers no loading control to ensure equal sample loading. Immunoblot is 100–1000-fold less sensitive, but allows for the probing of a sample loading control such as β-actin to ensure accurate protein loading. In this report, we describe two simple protocols that combine gelatin zymography to study MMP-2 and -9 levels with an in-gel β-actin immunoblot loading control, thus combining sensitivity and accuracy in a single assay. The protocols incorporate the loading of molecular weight markers to demarcate MMP-2/-9 from the β-actin. The first protocol utilizes the overlay of a 10% zymography gel over a 5% Tris-Glycine separating gel from which the β-actin is transferred. The second protocol involves the direct transfer of the β-actin from a single 10% zymography gel.

## INTRODUCTION

Matrix metalloproteinases (MMPs), particularly MMP-2 and -9, have been widely studied in many physiological processes and pathologies including tissue remodeling, angiogenesis, platelet aggregation, immunity, inflammation, arthritis, cardiovascular disease, and cancer. One of the most widely utilized techniques to measure MMP-2 and -9 in physiological fluids, cells, and tissues has been gelatin zymography. Since first described by Heussen and Dowdle, initially to study plasminogen activators [1], it has become the cornerstone assay for most studies into MMP-2 and -9 function. Zymography incorporates non-reducing sulfate polyacrylamide gel electrophoresis for separation of proteins and copolymerized substrate for detection of in gel enzymatic activity, with gelatin being the preferred substrate for MMP-2 and -9 (gelatinases A and B, respectively). It has exquisite sensitivity capable of detecting picogram quantities of both the zymogen and activated forms of these two MMPs [2]. The one shortcoming of zymography is the absence of an in-gel loading control to detect and correct for any errors in sample/protein loading. Hence, MMP-2 and -9 immunoblotting followed by immunoblotting for a housekeeping protein such as β -actin is often performed instead of or in addition to gelatin zymography [3].

Although antibodies to MMP-2 and -9 are widely commercially available, immunoblot can be a technically challenging technique that may require considerable time and effort to establish various assay conditions such as type of electrophoretic transfer, blocking, and/or antibody concentrations. Moreover, immunoblot is considerably less sensitive (detects nanogram quantities of protein) compared to zymography, even when utilizing polyclonal antibodies. This limitation may be of great importance in instances when there is a limited amount of starting sample to work with, such as when analyzing human platelets or rare clinical samples.

Hence, the purpose of our study was to develop a zymography-based assay sensitive to detect picogram quantities of MMPs and one that would allow for the correction of sample loading errors by incorporating an intra-gel loading control.

## MATERIALS AND METHODS

### Reagents

Unless otherwise indicated all chemicals were obtained from Sig ma-Aldrich (Oakville, ONT, Canada). Recombinant 72 KDa human pro-MMP-2 (rhMMP-2) and goat polyclonal anti-human MMP-2 antibody were obtained from R&D Systems (Minneapolis, MN, USA). Anti-human β -actin HRP-conjugated antibody was obtained from Sigma-Aldrich. Precision Plus Protein Dual Color molecular weight standards were obtained from Bio-Rad (Mississauga, ONT, Canada). Polyvinylidene fluoride (PVDF) membrane was obtained from EMD Millipore (Billerica, MA, USA) .

### Platelet isolation

Approval for the current study was obtained from the University of Alberta Health Research Ethics Board. Following written informed consent, from healthy volunteers, prostacyclin-washed platelets were prepared as described previously [4, 5].

### Gel preparation

Approach one: Utilizing the Bio-Rad Mini-Protean Tetra Cell mini gel (0.75 mm thickness) system, short and spacer plates were assembled into casting frames. A line was demarcated with a marker one-third (approximately 2 cm) from the bottom of the short plate. A 5% polyacrylamide Laemmli-based Tris-Glycine separating gel was poured up to the demarcating line, followed by a 0.5 cm layer of 95% ethanol. The gel was allowed to polymerize for approximately 45 min at room temperature. After complete polymerization of the 5% gel, the 95% ethanol was decanted. Next, a 10% Tris-Glycine separating gel incorporating gelatin (2 mg/ml) as substrate was overlaid to within 4 mm of well combs. Once again, a thin layer of 95% ethanol was overlaid and the gel was allowed to polymerize at room temperature. Upon polymerization the 95% ethanol was decanted and a 4% polyacrylamide stacking gel poured and allowed to polymerize around 10 well combs.

Within the second approach a standard 10% Tris-Glycine separating gel incorporating gelatin (2 mg/ml) as substrate was poured, allowed to polymerize at room temperature, and then a 4% polyacrylamide stacking gel poured and allowed to polymerize around 10 well combs as described above.

### Running of gels

**Figure 1:**
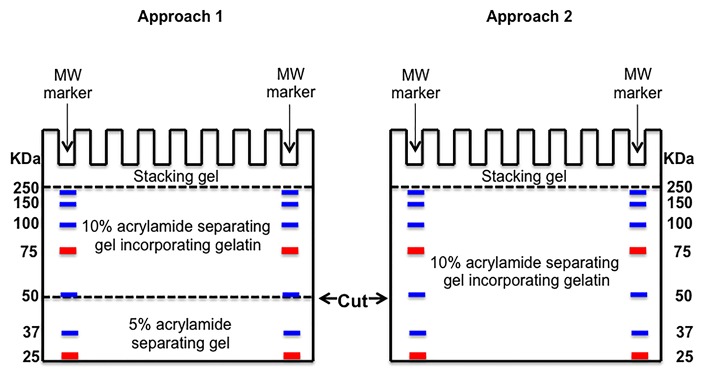
**A schematic demonstrating when to stop the gel run and where to cut the gels to obtain the β-actin loading control.**

Dual color molecular weight standards (10 μl) were loaded into the first and last wells of gels, while recombinant human MMP-2 standards (10 ng–10 pg) and human platelet lysate samples (1–10 μg/20 μl, non-reduced) were loaded in wells 2 through 9. The gels were run in standard Tris-Glycine-SDS running buffer at 4°C at a constant 150 V. Within approach one, the gels were run until the 50 KDa molecular weight marker reached the top of the 5% acrylamide separating gel. At this point the run was stopped and electrophoresis cell disassembled. Using a short ruler a cut was made at the 50 KDa MW marks to detach the gelatin-zymography from the 5% acrylamide immunoblot separating gel (**Fig. 1**). Within the second approach, the gels were run until the 50 KDa molecular weight marker reached the top of the bottom one-third of the short plate. As above, at this point the run was stopped and electrophoresis cell disassembled. Using a short ruler a cut was made at the 50 KDa MW marks generating a portion of the gel containing MMP-2 and -9 (> 50 KDa portion) and a gel containing β -actin (< 50 KDa portion) (**Fig. 1**).

### MMP and β-actin detection

The gelatin-incorporated gel from approach one and > 50 KDa containing gel from approach two were next processed according to standard zymography methodology. The gels were washed 3 × 20 min in 2% Triton X-100, followed by 2 washes of 20 min in 50 mM Tris-HCl zymography incubation buffer pH 7.6 supplemented with 150 mM NaCl, 5 mM CaCl_2_ and 0.05% NaN_3_. Finally, the gels were incubated in zymography incubation buffer at 37°C for 24–48 h, then stained in 0.05% Coomassie Brilliant Blue in 25% methanol and 10% acetic acid and destained in 20% isopropanol with 10% acetic acid. Bands of gelatinolytic activity were detected and documented using a Bio-Rad VersaDoc MP5000.

The 5% separating gel (approach one) and < 50 KDa portion (approach two) were then soaked for 10 min in transfer buffer (25 mM Tris-base, 192 mM glycine, 0.05% SDS) and then transferred onto PVDF membrane using a Bio-Rad semi-dry transfer system (25 V, 30 min). Following transfer PVDF membrane was blocked overnight in 5% non-fat dry milk and then incubated with anti-β-actin HRP conjugated antibody (1:40000) at room temperature for 30 min. The membrane was washed for 20 min with four changes of T-TBS and developed with ECL Prime (Amersham). Chemiluminescence was detected using a VersaDoc MP5000.

### Immunoblot

MMP-2 immunoblots were performed under denaturing and reducing conditions as described previously utilizing a 8% polyacrylamide Laemmli-based Tris-Glycine separating gel [3].

### Statistics

Statistics were performed using Graph Pad Prism 3.0. All means are reported with SE.

## RESULTS

**Figure 2:**
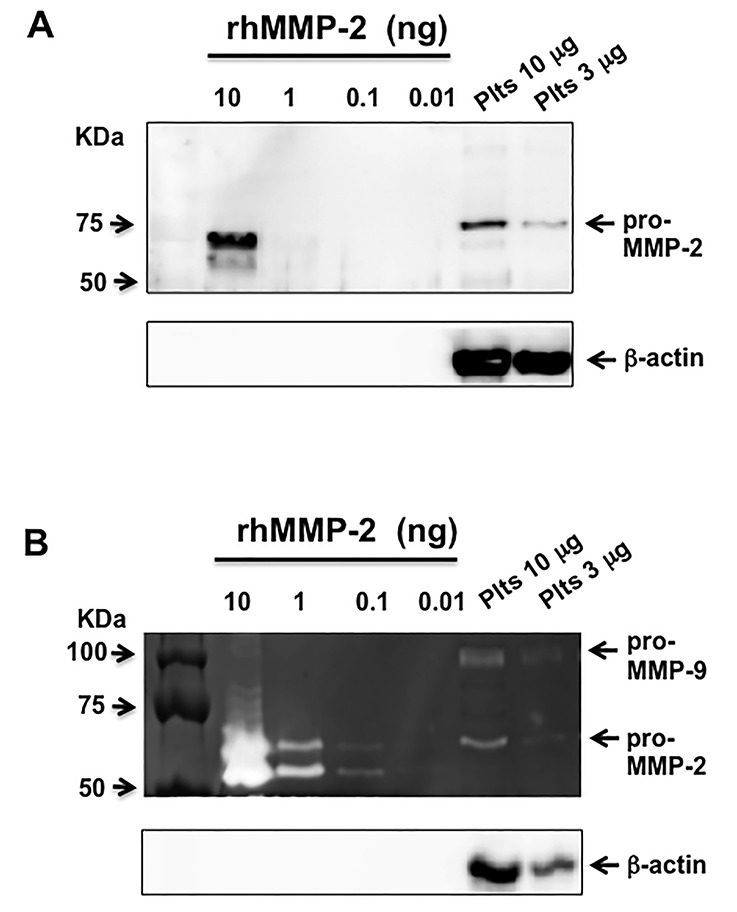
**A comparison of the sensitivity of immunoblot to loading-controlled zymography**. **A**. A representative MMP-2 immunoblot along with a β-actin loading control. **B**. A representative gelatin zymogram of rhMMP-2 and human platelet (Plts) samples. The zymogram incorporated a 5% separating gel that was utilized for the transfer and detection of β-action as a loading control. Representative gels are from an N = 3 experiments.

**Figure 3:**
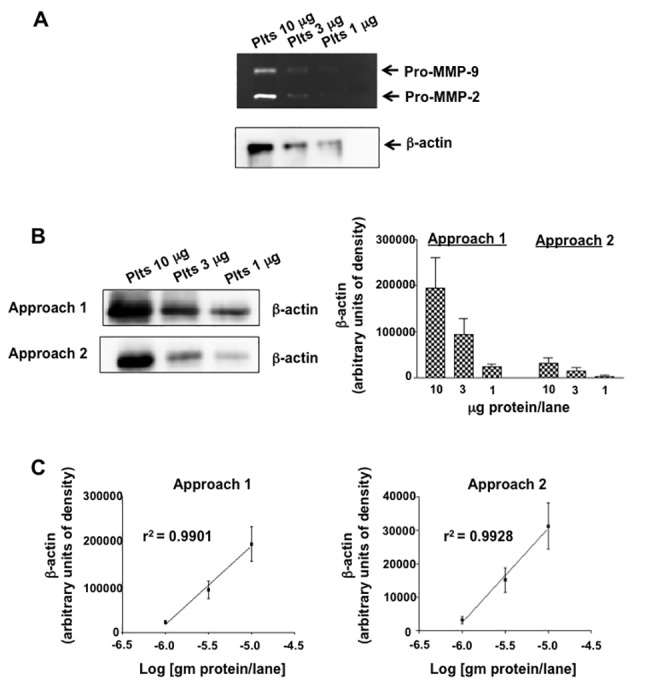
**A comparison of the transfer efficiency of loading-controlled zymography protocol 1 *vs .* protocol 2**. **A**. A representative gelatin zymogram of human platelet samples. The zymogram incorporates a β-actin loading control that was obtained from the direct transfer of < 50KDa portion of the zymogram on to PVDF. **B** and **C**. Comparisons of extent and linearity of the transfer of β-actin from the 5% separating gel (approach 1) to that directly from the < 50KDa portion of the zymogram (approach 2).

Utilizing a polyclonal anti-MMP-2 antibody immunoblot detected 10 ng of recombinant human pro-MMP-2 (rh-pro-MMP-2) standard (**Fig. 2A**). The zymogram in approach one detected 0.01–0.01 ng of rhMMP-2 activity, a 1000-fold increase in sensitivity compared to the immunoblot (**Fig. 2B**). The benefit of the β-actin loading control in the zymogram allowed for the confirmation that decreased pro-MMP-2 gelatinolytic activity in the 3 μg platelet protein lane compared to the 10 μg lane was indeed due to a difference in protein loading and not intrinsic activity of the sample. Similarly, direct transfer of β -actin from the < 50 KDa portion of a zymogram (approach 2) also allowed for a loading control (**Fig. 3A**). A comparison of the transfer of β-actin from the 5% acrylamide separating gel compared to the transfer directly from the zymogram revealed that transfer from the 5% gel is more efficient as 6-fold more β-actin was detected upon immunoblotting (**Fig. 3B**). Although less efficient, direct transfer of β-actin from the zymogram demonstrated the same linearity in β-actin detection over the 1–10 μg protein per lane range as transfer from the 5% separating gel (**Fig. 3C**).

## DISCUSSION

We introduce two simple methods that combine gelatin zymography to study MMP-2 and -9 levels with an in-gel β -actin immunoblot loading control, thus combining sensitivity and accuracy in a single assay. Utilizing these two methods, within platelet samples, pro-MMP-2 was identified in the gelatin-containing zymography portions of the gels as bands matching the gelatinolytic activity of recombinant pro-MMP-2 (72 KDa– top band). *In situ*, following cell surface binding, pro-MMP-2 is activated by membrane-type-1-matrix metalloproteinase MMP (MT1-MMP) [6, 7]; however, *in vitro* at high concentrations pro-MMP-2 may undergo autolytic activation into active form [8]. This autolytically activated form was detected within the recombinant human MMP-2 standards as the lower band in both immunoblot and loading-controlled zymography and facilitated identification of the pro-form of MMP-2 in human platelets. Importantly, zymography detects both active and pro-forms of zymogens. This ability to detect pro-MMPs is due to the SDS within the gels during running which causes an unfolding of the enzymes exposing the catalytic site even in the presence of the inhibitory propeptide domain [9]. The inhibitory propeptides are only partially refolded during the Triton X-100 and incubation buffer washes, and as a consequence the in-gel proteolytic activity of the pro-MMPs does not represent *in situ*/*in vivo* activity. Similarly, although tissue inhibitors of matrix metalloproteinases (TIMPs) normally tightly regulate active MMPs (such as MMP-2) *in vivo* [10, 11], TIMPs dissociate from MMPs during gel running and the in-gel proteolytic activity of active-isoform MMPs represents potential *in situ*/*in vivo* activity. Hence, gelatin zymography measures total MMP-2 and/or -9 levels with high (pg) sensitivity and not their *in vivo* activity *per se*.

Although 100–1000-fold more sensitive than immunoblot in detecting MMP levels, confirmation of equal or unequal protein loading is not possible with traditional zymography alone. Therefore, we developed two protocols that incorporate a β -actin loading control to overcome this important zymography limitation. In addition, β -actin loading-controlled zymography also simultaneously detected pro-MMP-9 activity, another advantage over immunoblot which depending on the antibody only identifies either MMP-2 or -9. In instances in which sample protein content may be a limiting factor, approach one utilizing a 5% separating gel may be advantageous to perform, as more of β -actin would be transferred. Conversely, the direct transfer of β -actin from the < 50KDa portion of a zymogram is simpler and quicker to perform as it does not require the preparation of an initial 5% separating gel. Irrespective of the approach, for improved accuracy, we recommend the incorporation of a β -actin loading control for all gelatin zymography applications investigating pro- or active MMP-2/-9.

## Abbreviations used

MMPs: matrix metalloproteinases
MT1-MMP: membrane-type-1-matrix metalloproteinase MMP
PVDF: polyvinylidene fluoride
rhMMP-2: recombinant 72 KDa human pro-MMP-2
